# Energy and nutrient intakes among Sri Lankan adults

**DOI:** 10.1186/1755-7682-7-34

**Published:** 2014-07-11

**Authors:** Ranil Jayawardena, Shalika Thennakoon, Nuala Byrne, Mario Soares, Prasad Katulanda, Andrew Hills

**Affiliations:** 1 Institute of Health and Biomedical Innovation, Faculty of Health, Queensland University of Technology, Brisbane, Queensland, Australia; 2 Diabetes Research Unit, Faculty of Medicine, University of Colombo, Colombo, Sri Lanka; 3 Curtin Health Innovation Research Institute, School of Public Health, Faculty of Health Sciences, Curtin University, Perth, WA, Australia; 4 Centre for Nutrition and Exercise, Mater Research Institute–The University of Queensland, Brisbane, Australia

**Keywords:** Dietary survey, Nutrition survey, Energy intake, Sri Lanka, Adults

## Abstract

**Introduction:**

The epidemic of nutrition related non-communicable diseases such as type 2 diabetes mellitus and obesity has reached to epidemic portion in the Sri Lanka. However, to date, detailed data on food consumption in the Sri Lankan population is limited. The aim of this study is to identify energy and major nutrient intake among Sri Lankan adults.

**Methods:**

A nationally-representative sample of adults was selected using a multi-stage random cluster sampling technique.

**Results:**

Data from 463 participants (166 Males, 297 Females) were analyzed. Total energy intake was significantly higher in males (1913 ± 567 kcal/d) than females (1514 ± 458 kcal/d). However, there was no significant gender differences in the percentage of energy from carbohydrate (Male: 72.8 ± 6.4%, Female: 73.9 ± 6.7%), fat (Male: 19.9 ± 6.1%, Female: 18.5 ± 5.7%) and proteins (Male: 10.6 ± 2.1%, Female: 10.9 ± 5.6%).

**Conclusion:**

The present study provides the first national estimates of energy and nutrient intake of the Sri Lankan adult population.

## Introduction

The epidemic of nutrition related non-communicable diseases (NCDs) such as type 2 diabetes mellitus, obesity, Cardio Vascular Diseases (CVDs) and certain cancers are continuing to challenge the health sectors in Asia [1]. Sri Lanka is a low-middle income South Asian country with a population of 20 million. Despite Sri Lanka’s relatively good health status, during the last two decades NCDs have become a more prominent health issue in the country [2]. A quarter of Sri Lankan adults suffer from metabolic syndrome [3]. According to Sri Lanka Diabetes and Cardiovascular Study (SLDCS), the prevalence of diabetes among Sri Lankan adults was nearly 11% and one fifth of adults in Sri Lanka have diabetes or pre-diabetes while one third of those with diabetes are undiagnosed [4]. Premarathna *et al.,* have also reported that there was an increase in the incidences of hospitalization of Sri Lankan adults by 36%, 40% and 29% due to diabetes mellitus, hypertensive disease and ischemic heart disease, respectively, in 2010 compared to 2005 [5]. In Sri Lanka, diet-related chronic diseases currently account for 18.3% of all deaths and 16.7% of hospital expenditure [1]. There is a significant health burden due to NCDs and this will be a challenge to the health sector in a developing country like Sri Lanka.

Some methods to assess the quantity and quality of dietary intake include prospective food records (with weighed or estimated food portions), retrospective 24-hour recalls (24 HDR), and food frequency questionnaires (FFQs) [6]. The 24HDR which is less time consuming and has a low respondent burden, is the method used to gather the quantitative estimate of all foods and beverages that an individual has consumed in the previous 24 hours at a population level. Several national dietary surveys have used 24 HDR and it is known to be acceptable for gathering dietary information on a given day at the population level [7,8].

National diet and nutrition surveys provide valuable information on a possible partial explanation for the eople’s health status and disease risk [9]. Assessment of the dietary and nutritional status of the population is essential to monitor the ongoing nutrition transition in a country [6]. As a developing country, no studies have been carried out to investigate the information on the diet of Sri Lankans and their nutritional status at a national level. Since Sri Lanka is a multi cultural country, peoples’ foods and dietary habits at a national level should be assessed with a representative sample of Sri Lankan adults, which will be more useful to implement health policies and to initiate many interventions. By keeping this view in mind, the current dietary survey was carried out to assess the intakes of energy, macro-nutrients and selected other nutrients with respect to socio demographic characteristics and the nutritional status of Sri Lankan adults.

## Methodology

### Study sampling and the subjects

The eligible respondents of this study were healthy Sri Lankan adults aged ≥ 18 years recruited from a sub sample of a Sri Lanka Diabetes and Cardiovascular Study [4]. In this study, a total of 600 subjects were randomly selected representing all nine provinces. This sample population was then stratified for area of residence and ethnicity. Description of sample selection is published elsewhere [10]. Written informed consent for participation in the study was obtained and ethical approval for this study was taken from the Ethical Review Committee, Faculty of Medicine, University of Colombo, Sri Lanka.

### Measurements

#### Socio-demographic variables

The selected subjects were initially contacted via telephone or a postal notice by the study team and the information regarding the study was provided in order to obtain their willingness to participate in the study. On the study day, the purpose of the study was briefly explained to the subjects and the information sheets of the study were also given out. Written consent was obtained from each volunteer prior to data collection. Socio-demographic details and diabetes status were obtained by using an interviewer-administered questionnaire and body weight and height were measured using a standard method. Areas of residence, ethnicities, and education levels were categorized according to Sri Lankan governmental standards [11]. Body mass index (BMI) was calculated by weight (in kilograms) divided by height squared (in meters) and several cut-offs were presented as recommended by WHO experts for Asian populations [12].

#### Dietary assessment

Dietary data were obtained from a 24 HDR method. The subjects were asked to recall all foods and beverages, consumed over the previous 24-hour period. Respondents were probed for the types of foods and food preparation methods. For uncommon mixed meals, the details of recipes and preparation methods were collected at the time of taking the 24 HDR. Dietary recalls were collected by two trained nutritionists who had received uniform training and adhered to the standard operating procedure (SOP). As dietary assessment aids, the standard household measurements such as plate, bowl, cup, glass, and different spoons etc. and food photograph atlases were used to facilitate the quantification of portion sizes. One medium sized coconut spoon of rice was taken as 100 g, a full plate as 400 g, one cup of liquid as 150 ml, one glass of liquid as 200 ml, a table spoon as 15 g and a tea spoon was taken as 5 g. For different curries, weights of average respective amounts were taken. Household measurements were clarified by demonstration of the real utensils and the food portion size photographs. When subjects recalled some food amount in grams, that information was directly entered. Further details of dietary assessment were published previously [10].

### Data analysis

All foods recorded in 24 HDR were converted into grams and then, the intake of total energy, macro nutrients (Carbohydrate, Protein and Fat), sodium and dietary fiber were analyzed using NutriSurvey 2007 (EBISpro, Germany) which was modified for Sri Lankan food recipes. As no updated nutritional database has been gathered for some Sri Lankan food, we used the US Department of Agriculture (USDA) nutrient database [13] as our standard to estimate nutrient content in addition to local and regional food composition databases [14,15]. Due to the absence of energy and nutrient information on local mixed cooked dishes, we used a cookery book [16]. All the recipes were accepted after checking for face validity by consulting local housewives and nutritionists. According to recipes, ingredients were weighed to the nearest 1 g for edible portions of the foods. Then food items were cooked accordingly and the end product was weighed. Nutritional composition of the final meal was calculated by entering nutritional values and the weights of individual ingredients to the spreadsheet. The sum of each nutrient was computed and standardized to 100 g of final product. We also excluded participants whose reported daily energy intake was not between 800 and 4200 kcal to identify under- and over-reporters of food intake [17].

### Statistical analysis

All data were doubly entered and rechecked in Microsoft Excel 2007. Data sorting and cleaning were carried out before data analysis. Data on energy, macro-nutrients and some selected nutrient intakes were transferred from the NutriSurvey 2007 to the Minitab version 15.0 for statistical analysis. Nutrient intake distributions are presented as mean ± SE, median, 25th and 75th percentiles to characterize population intake levels for socio-demographic characteristics (gender, ethnicity, age groups, and educational levels) and BMI categories. One-way ANOVA and t-test were used to examine the differences in mean intakes energy and nutrients intakes. P value < 0.05 was considered statistically significant.

## Results

### Socio-demographic profile

From 600 subjects, 491 (81.8%) participated and 28 of subjects under-reported their energy intake. So, a total of 463 (77.2%) was included for the analysis. Socio demographic profiles and BMI categories of the subjects are presented in Table 1. The majority of the subjects were from rural areas (59.7%) and 33% of the population were from urban areas followed by the estate sector (tea plantation area) 7.3%. The majority were women (n = 297). By ethnic groups, Sinhalese (78%), Sri Lankan Tamil (9%), Indian Tamil (7%), and Muslim (6%) in this survey. Adults between the age of 41 and 50 years formed the biggest group (25.27%) while the smallest group was the youngest adults aged between 18-30 yrs (13.17%). It was significant that a majority of the study population (39%) had received formal education up to Ordinary Level. The next largest group was adults (25%) who had studied up to Advanced Level.

**Table 1:** Socio-demographic characteristics of the survey population

**Characteristics**	**Total (n = 463)**		**Men (n = 166)**		**Women (n = 297)**	
	**n**	**%**	**n**	**%**	**n**	**%**
**Area of residence**						
Urban	153	33.0	45	26.5	108	36.4
Rural	276	59.6	102	61.4	174	58.6
Estate	34	7.4	19	11.5	15	5.0
**Age group (yrs)**						
18-29	61	13.2	27	16.3	34	12.7
30-39	84	18.1	23	13.8	61	22.8
40-49	117	25.3	38	22.9	79	29.6
50-59	106	22. 9	40	20.1	66	24.7
>60	95	20.5	38	22.9	57	21.4
**Ethnicity**						
Sinhala	360	77.7	118	71.0	242	82.5
Muslim	27	5.8	8	4.8	19	6.4
Sri Lankan Tamil	42	9.1	20	12.1	22	7.4
Indian Tamil	34	7.3	20	12.1	14	4.7
**Educational level**						
No schooling	27	58.3	11	6.6	16	5.4
Up to 5 years	113	24.4	43	25.9	70	23.6
Up to O/L	182	39.3	59	35.5	123	41.4
Up to A/L	116	25.1	46	27.7	70	23.6
Graduate	25	5.4	07	4.2	18	6.1
**BMI category**						
≤ 18.5 kg.m^-2^	64	13.8	29	17.5	35	11.8
> 18.5 - ≤ 22.9 kg.m^-2^	163	35.2	75	45.2	88	29.6
> 23 - ≤ 24.99 kg.m^-2^	76	16.4	21	12.6	55	18.5
> 25 - ≤ 27.5 kg.m^-2^	95	20.5	32	19.3	63	21.1
≥ 27.5 kg.m^-2^	65	14.1	09	5.4	56	18.9

### Energy intake

Table 2 represents the distribution of energy intake of Sri Lankan adults. The mean energy intake of men was significantly higher (1912.7 kcal/d) than that of women (1513.6 kcal/d) (p < 0.05). People living in the estate sector have a significant lower energy intake compared to both the urban and rural subjects (p < 0.05). Muslims had the highest intake of daily energy (1748.8 kcal) while Indian Tamils had the lowest (1437.7 Kcal/d) which statistically significant for both men and women (p < 0.05). Energy consumption of both gender groups declined gradually with their age. Energy intake increased gradually with educational level. According to BMI categories, lower energy levels were reported in both extremes and no distinct pattern was seen.

**Table 2:** Energy intake (kcal) of Sri Lankan adults by socio-demographic characteristics

**Characteristics**	**All subjects (n =463)**					**Men (n = 166)**					**Women (n = 297)**				
	**Mean**	**±SE**	**Median**	**Percentiles**		**Mean**	**±SE**	**Median**	**Percentiles**		**Mean**	**±SE**	**Median**	**Percentiles**	
				**25**	**75**				**25**	**75**				**25**	**75**
**Area of residence**															
Urban	1669	45	1594	1217	2005	1910	89	1899	1522	2218	1569	50	1453	1158	1885
Rural	1677	32	1590	1304	1994	1975	57	1926	1518	2300	1502	32	1462	1193	1728
Estate	1439	61	1468	1114	1690	1581	72	1635	1294	1847	1258	87	1340	973	1470
**Ethnicity**															
Sinhala	1669	28	1256	1589	1977	1947	51	1901	1518	2247	1533	30	1447	1173	1790
Muslim	1749	84	1435	1647	2156	1949	173	1984	1458	2324	1664	91	1626	1401	2026
Sri Lankan Tamil	1671	100	1189	1526	2091	2061	161	2094	1660	2352	1317	62	1334	1071	1523
Indian Tamil	1438	61	1468	1114	1690	1546	77	1634	1225	1833	1283	90	1354	993	1472
**Age group (years)**															
18-30	1832	75	1942	1297	2301	2166	95	2064	1942	2392	1567	91	1385	1108	2052
31-40	1808	64	1661	1403	2059	2250	148	1777	1633	2726	1641	56	1596	1346	1892
41-50	1634	46	1545	1268	1906	1810	89	1848	1418	2099	1549	51	1507	1197	1821
51-60	1614	49	1544	1233	1905	1859	70	1639	1595	2037	1465	60	1361	1134	1701
>61	1487	47	1394	1138	1747	1688	84	2155	1305	2094	1353	48	1257	1068	1626
**Educational level**															
No schooling	1287	73	1202	905	1589	1442	115	1484	1123	1792	1181	89	1117	882	1469
Up to 5 years	1556	39	1528	1233	1831	1748	69	1715	1380	1992	1438	42	1451	1138	1655
Up to O/L	1677	40	1788	1299	2468	1970	77	1873	1493	2356	1536	41	1473	1194	1787
Up to A/L	1823	55	1763	1378	2183	2058	89	2086	1590	2292	1668	65	1583	1224	2008
Graduate	1594	102	1470	1226	2000	2221	119	2234	1977	2543	1350	78	1265	1065	1635
**BMI category**															
≤ 18.5 kgm^-2^	1548	64	1409	1173	1799	1782	113	1637	1288	2151	1354	54	1325	1135	1466
>18.5 - ≤ 22.9 kgm^2^	1731	45	1642	1296	2064	1946	66	1886	1522	2290	1548	56	1439	1113	1907
>23 - ≤ 24.9 kgm^-2^	1666	60	1570	1294	1857	1910	118	1817	1493	2083	1532	62	1466	1233	1724
> 25 - ≤ 27.5 kgm^-2^	1674	52	1677	1285	1977	1988	99	1987	1650	2324	1556	56	1579	1224	1790
≥ 27.5 kgm^-2^	1541	54	1520	1169	1871	1851	147	1892	1569	2103	1491	56	1430	1138	1728

### Carbohydrate intake

**Figure 1:**
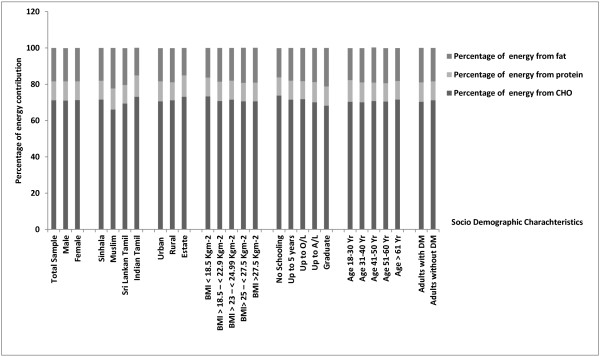
Percentage energy contribution from macronutrients according to gender, ethnicity and area of residance, BMI, educational level and age group.

The mean daily carbohydrate intake was shown in Table 3. The total mean carbohydrate intake of Sri Lankan adults was approximately 304.4 g (71.2% of total energy from Carbohydrates as shown in Figure 1). By strata, rural adults had a higher intake of carbohydrate (307.7 g) than their estate counterparts (270.3 g). Mean carbohydrate intake was highest in Sinhalese (308.7 g) and lowest in Indian Tamils (269.9 g). Male adults’ carbohydrate intake (352.4 g/day) was significantly higher than that of women (277.5 g/day). Carbohydrate intake declined with age.

**Table 3:** Carbohydrate intake (g) of Sri Lankan adults by socio-demographic characteristics

**Characteristics**	**All subjects (n =463)**					**Men (n = 166)**					**Women (n = 297)**				
	**Mean**	**±SE**	**Median**	**Percentiles**		**Mean**	**±SE**	**Median**	**Percentiles**		**Mean**	**±SE**	**Median**	**Percentiles**	
				**25**	**75**				**25**	**75**				**25**	**75**
**Area of residence**															
Urban	305.9	8.7	290.7	217.5	373.5	343.5	16.3	346.7	262.8	414.1	290.4	10.0	259.1	259.1	349.1
Rural	307.7	6.2	292.3	233.0	365.7	367.1	11.4	353.9	285.3	425.6	272.8	5.88	262.1	262.1	324.7
Estate	270.3	11.8	266.6	213.0	320.6	295.0	13.7	309.5	237.2	345.4	239.0	17.7	237.2	237.2	262.7
**Ethnicity**															
Sinhala	308.7	5.6	292.3	229.7	368.0	363.1	10.3	346.8	289.3	427.7	282.2	5.9	262.0	214.3	330.8
Muslim	298.0	13.9	299.9	245.2	348.4	316.8	30.7	282.8	247.3	404.6	290.0	15.1	299.9	245.2	348.4
Sri Lankan Tamil	298.9	17.7	269.9	203.4	375.1	367.9	27.3	369.6	315.9	402.0	236.1	12.6	226.8	199.6	267.2
Indian Tamil	269.9	11.9	266.6	213.0	320.6	288.0	14.7	300.6	233.8	341.5	244.2	18.1	237.7	196.2	270.8
**Age group (years)**															
18-30	338.9	15.1	340.2	233.7	425.8	401.9	19.0	400.7	345.5	440.1	289.0	18.6	247.7	206.0	392.2
31-40	305.0	10.8	299.5	252.8	344.1	423.6	28.6	395.8	309.5	477.8	305.0	10.8	299.5	252.8	344.1
41-50	298.7	8.6	294.6	232.8	352.1	330.4	17.1	316.3	252.4	381.3	283.4	9.4	272.6	226.9	333.3
51-60	291.9	9.4	273.5	225.5	348.5	339.1	13.2	329.0	275.1	398.1	263.2	11.4	235.4	198.2	321.7
>61	273.8	9.1	261.0	203.9	324.8	310.2	16.4	306.6	233.7	371.9	249.5	9.35	239.0	201.4	390.4
**Educational level**															
No schooling	242.9	13.5	235.7	174.2	305.5	270.9	21.1	259.2	211.4	318.2	223.6	16.4	216.2	160.6	258.0
Up to 5 years	286.4	7.8	276.4	228.9	331.6	323.8	14.3	317.6	244.5	386.8	263.5	7.9	261.3	216.1	307.1
Up to O/L	309.3	7.7	290.8	233.2	364.8	366.8	15.5	345.5	290.9	415.5	281.8	7.4	262.6	221.1	329.5
Up to A/L	332.5	11.1	323.1	243.7	399.0	373.1	16.9	374.7	300.3	427.9	305.9	13.8	279.6	216.0	360.8
Graduate	284.8	18.5	239.0	203.6	343.1	399.2	22.5	401.6	323.0	440.4	240.3	13.5	232.2	200.0	293.1
**BMI category**															
≤ 18.5 kgm^-2^	292.0	13.3	254.2	220.1	329.8	342.6	23.5	323.7	237.5	401.9	250.1	10.3	238.2	205.7	268.8
>18.5 - ≤ 22.9 kgm^2^	318.1	8.7	301.6	230.5	376.3	356.3	12.5	335.8	291.5	418.3	285.5	11.0	258.5	213.2	349.5
>23 - ≤ 24.9 kgm^-2^	305.2	12.1	275.1	236.2	349.9	350.5	22.6	346.7	266.6	401.7	280.2	12.8	258.5	213.2	349.5
> 25 - ≤ 27.5 kgm^-2^	303.4	9.8	301.9	236.3	356.3	363.2	20.3	368.0	291.3	426.0	280.9	9.8	283.1	214.9	331.7
≥ 27.5 kgm^-2^	282.4	10.4	264.6	221.4	334.1	325.8	28.2	317.6	263.4	378.0	275.5	11.0	262.8	211.1	329.4

### Protein intake

Sri Lankan adults recorded a mean daily protein intake of 44.6 g whilst men’s intake (52.8 g) was significantly higher than women’s intake (40.0 g). As shown in Table 4, rural (42.9 g/day) and estate (43.7 g/day) adults had similar daily intakes of protein. However, by ethnicity, mean protein intake was significantly higher in Muslims (52.2 g) compared others. Youngest group by age also consumed significantly more protein than others but only for men.

**Table 4:** Protien intake (g) of Sri Lankan adults by socio-demographic characteristics

**Characteristics**	**All subjects (n =463)**					**Men (n = 166)**					**Women (n = 297)**				
	**Mean**	**±SE**	**Median**	**Percentiles**		**Mean**	**±SE**	**Median**	**Percentiles**		**Mean**	**±SE**	**Median**	**Percentiles**	
				**25**	**75**				**25**	**75**				**25**	**75**
**Area of residence**															
Urban	47.8	3.6	41.0	31.8	53.3	62.7	11.6	47.7	37.8	67.1	41.6	1.4	38.8	29.7	50.5
Rural	42.9	0.9	39.8	32.5	50.7	48.7	1.6	45.6	35.6	57.9	39.5	1.0	37.6	29.9	46.2
Estate	43.7	2.4	42.1	32.6	54.9	50.4	3.1	53.0	38.8	61.9	35.1	2.2	33.8	27.3	44.4
**Ethnicity**															
Sinhala	44.2	1.6	39.8	32.1	50.5	52.6	4.5	35.6	35.6	57.1	40.1	0.9	37.5	29.7	47.6
Muslim	52.2	2.6	49.9	40.9	61.3	58.6	5.1	47.3	47.3	70.1	49.4	2.9	47.7	40.2	60.8
Sri Lankan Tamil	44.1	3.2	38.8	29.8	52.8	54.8	5.5	38.6	38.6	65.1	34.4	1.8	34.0	27.9	39.6
Indian Tamil	43.4	2.5	42.1	32.6	54.9	48.9	3.3	38.2	38.2	61.0	35.3	2.5	33.9	27.3	44.4
**Age group (yrs)**															
18-30	57.4	8.6	46.8	34.3	60.6	74.9	18.8	52.3	43.8	74.9	43.4	2.6	41.0	31.8	53.9
31-40	47.6	2.0	42.9	34.5	52.6	59.5	4.8	53.3	41.5	72.5	43.1	2.0	40.4	32.6	47.0
41-50	42.6	1.2	41.0	32.7	50.5	46.5	2.3	44.9	35.5	54.3	40.8	1.4	49.9	37.9	69.8
51-60	41.9	1.5	38.1	29.6	50.8	48.4	2.5	48.2	37.0	56.2	38.0	1.7	34.6	27.3	46.2
>61	39.1	1.4	34.4	29.9	45.4	43.7	2.5	40.2	32.2	54.9	36.0	1.6	33.5	28.6	41.0
**Educational level**															
No schooling	33.1	2.0	33.8	25.3	38.3	67.7	22.1	52.3	43.8	74.9	31.9	2.7	32.4	24.2	41.1
Up to 5 years	41.9	1.4	38.8	30.5	49.3	59.5	4.8	53.3	41.5	72.5	38.8	1.5	37.7	28.6	44.7
Up to O/L	42.7	1.1	39.6	32.4	50.5	46.5	2.3	44.9	35.5	54.3	39.4	1.1	36.4	30.0	46.3
Up to A/L	52.9	4.7	45.5	35.7	56.5	48.4	2.5	48.2	37.0	56.3	44.4	1.8	40.8	32.7	53.8
Graduate	44.24	3.5	40.2	32.2	57.5	43.7	2.5	40.2	32.2	54.9	39.4	4.1	34.3	29.5	41.9
**BMI Category**															
≤ 18.5 kgm^-2^	41.6	1.2	39.9	31.8	46.6	45.8	2.9	43.0	35.6	52.4	38.1	2.3	34.1	29.6	43.2
>18.5 - ≤ 22.9 kgm^2^	47.6	3.4	41.0	32.5	53.3	55.6	7.0	46.0	36.2	59.1	40.6	1.6	37.8	29.4	50.3
>23 - ≤ 24.9 kgm^-2^	44.6	2.0	41.1	32.7	49.3	52.8	3.9	47.7	35.6	59.0	40.0	1.8	34.1	29.6	43.2
> 25 - ≤ 27.5 kgm^-2^	43.8	1.5	39.9	32.6	54.4	52.6	2.8	53.2	41.9	64.4	40.5	1.7	37.9	30.5	48.7
≥ 27.5 kgm^-2^	41.1	1.7	37.7	29.5	48.3	52.1	6.2	56.3	33.3	70.8	39.3	1.7	36.5	29.0	46.3

### Fat intake

Estimated daily mean fat intake of Sri Lankan adults was 35 g. A more or less similar fat consumption was noted for rural and urban residents (Table 5) whereas estate people had significantly lower intake of fat (24.76 g; p < 0.05). The youngest age group recorded the highest fat intake (37.7 g) while the lowest intake was observed in the oldest age group (30.8 g). By ethnic groups, Muslims had the highest fat intake (44.7 g) whilst the Indian Tamils had the lowest (24 g) which is significantly lower than Muslims (p < 0.05). With education level, fat consumption was increased particularly among men. Adults with normal BMI and BMI > 25 - ≤ 27.5 kgm^-2^ had a higher fat intake than other BMI categories.

**Table 5:** Fat intake (g) of Sri Lankan adults by socio-demographic characteristics

**Characteristics**	**All subjects (n =463)**					**Men (n = 166)**					**Women (n = 297)**				
	**Mean**	**±SE**	**Median**	**Percentiles**		**Mean**	**±SE**	**Median**	**Percentiles**		**Mean**	**±SE**	**Median**	**Percentiles**	
				**25**	**75**				**25**	**75**				**25**	**75**
**Area of residence**															
Urban	35.3	1.3	31.3	23.8	43.2	42.8	2.98	37.1	27.6	58.2	32.2	1.3	29.8	22.8	38.5
Rural	36.1	0.9	34.2	23.8	43.8	41.9	1.70	39.1	29.6	50.2	32.7	1.1	30.2	21.6	40.6
Estate	24.8	1.8	22.3	17.0	34.8	27.3	2.39	22.8	17.9	35.4	21.6	2.6	18.7	14.6	26.4
**Ethnicity**															
Sinhala	34.8	0.8	32.4	23.0	42.6	40.4	1.53	37.6	28.7	49.8	32.1	0.9	29.8	21.5	39.6
Muslim	44.7	4.0	37.9	29.4	61.3	57.0	8.68	55.4	37.5	78.6	39.6	3.8	36.4	25.0	54.6
Sri Lankan Tamil	39.0	2.9	32.8	25.6	52.0	48.2	4.67	46.0	30.2	62.9	30.6	2.4	28.6	22.1	35.4
Indian Tamil	24.9	1.8	22.3	17.2	34.8	26.8	2.32	22.3	17.4	35.3	22.2	2.7	21.2	15.7	26.9
**Age group (years)**															
18-30	37.7	2.2	36.3	24.8	44.9	45.0	3.58	39.1	33.8	60.4	32.0	2.2	30.8	22.6	41.7
31-40	36.6	1.8	33.9	24.6	44.3	45.4	4.48	40.1	29.5	60.9	33.2	1.7	29.6	24.2	42.2
41-50	35.4	1.6	31.8	24.1	41.9	40.5	2.96	37.7	28.0	53.2	33.0	1.8	30.8	21.6	39.0
51-60	35.6	1.6	32.8	22.7	45.0	38.8	2.82	34.3	24.0	49.7	33.6	1.8	32.4	22.1	39.2
>61	30.8	1.4	27.4	20.7	39.6	36.1	2.39	34.2	23.7	46.8	27.3	1.7	24.4	19.0	33.0
**Educational level**															
No schooling	23.6	2.1	20.5	16.4	30.8	26.6	3.34	22.8	17.9	32.8	21.4	2.6	19.5	13.6	29.0
Up to 5 years	32.1	1.2	39.2	23.2	29.9	34.9	1.84	35.4	24.9	42.0	30.4	1.5	29.0	22.3	36.6
Up to O/L	35.0	1.2	31.7	22.1	44.8	40.3	2.30	38.0	25.3	52.2	32.5	1.3	29.0	20.7	41.0
Up to A/L	39.6	1.6	36.4	26.7	46.0	46.0	3.05	39.4	31.9	60.8	35.4	1.7	34.6	24.3	42.8
Graduate	39.3	3.9	34.2	23.8	58.5	61.6	5.25	60.9	56.6	76.1	30.6	3.2	35.4	21.8	28.4
**BMI category**															
≤ 18.5 kgm^-2^	28.8	1.8	24.2	17.3	35.6	33.3	3.04	28.0	19.6	43.8	25.1	2.0	22.6	16.4	30.6
>18.5 - ≤ 22.9 kgm^2^	37.2	1.4	34.8	23.7	46.7	42.6	2.16	38.8	29.2	51.8	32.3	1.5	30.0	22.1	40.9
>23 - ≤ 24.9 kgm^-2^	34.2	1.6	32.5	25.4	39.5	39.0	3.52	33.7	26.9	57.5	32.0	1.5	32.6	25.6	38.0
> 25 - ≤ 27.5 kgm^-2^	36.8	1.6	35.8	24.1	45.5	42.2	3.06	38.3	29.6	53.3	34.8	1.9	32.8	22.2	44.8
≥ 27.5 kgm^-2^	34.0	2.0	28.7	24.0	40.0	44.9	7.02	37.8	26.4	66.2	32.3	2.0	28.6	22.4	39.3

### Energy contribution from macro nutrients

As a whole, 71.2% energy come from carbohydrates among Sri Lankan adults, 10.8% from protein and 18.9% from fat. Comparisons of the percentage of energy derived from macronutrients according to socio demographic profile and BMI categories were shown in Figure 1. By ethnic distribution, Muslims had more energy from fat (22.3%) while Indian Tamils had the lowest amount of fat (15.5%) and highest intake of carbohydrates (75%). The percentage of calories from protein were relatively higher among the graduates. In contrast, adults who did not receive a formal education had a higher percentage of energy from carbohydrates compared to other groups. There was no difference in energy distribution between diabetic and non-diabetic subjects.

### Dietary fiber

The daily mean dietary fiber intake of Sri Lankan adults was 18.1 g (men: 21.3 g; women: 16.3 g; p < 0.05). By area of residence, estate adults had a higher dietary fiber intake (20.6 g) than their urban and rural counterparts (Table 6). Mean dietary fiber intake was highest in Indian Tamils (20.6 g) and lowest in Sinhalese (17.6 g) (p < 0.05). Dietary fiber intake increased with educational level and a similar trend was observed for women as men. Daily dietary fiber intake was always higher among men than women with different socio demographic characteristics. Adults aged > 60 years had the lowest intake of fiber.

**Table 6:** Dietary fiber intake (g) of Sri Lankan adults by socio demographic characteristics

**Characteristics**	**All subjects (n =463)**					**Men (n = 166)**					**Women (n = 297)**				
	**Mean**	**±SE**	**Median**	**Percentiles**		**Mean**	**±SE**	**Median**	**Percentiles**		**Mean**	**±SE**	**Median**	**Percentiles**	
				**25**	**75**				**25**	**75**				**25**	**75**
**Area of residence**															
Urban	18.1	0.7	16.2	12.2	22.6	19.7	1.4	17.0	13.8	25.5	17.5	0.8	15.4	11.9	21.0
Rural	17.7	0.5	16.6	12.2	21.3	21.3	0.8	18.6	15.6	26.8	15.6	0.5	15.1	11.1	19.0
Estate	20.6	1.9	17.7	12.8	28.7	24.9	2.8	22.3	14.6	33.1	15.2	1.5	16.7	8.8	19.5
**Ethnicity**															
Sinhala	17.7	0.4	16.4	12.1	21.4	20.2	0.8	17.8	14.1	24.9	16.4	0.5	15.6	11.3	19.7
Muslim	18.8	1.4	18.0	12.8	24.4	22.4	2.3	22.7	16.7	24.8	17.2	1.7	15.3	12.1	19.5
Sri Lankan Tamil	18.8	1.3	17.4	12.4	26.4	23.8	2.0	26.4	15.6	31.8	14.3	1.0	13.6	11.1	18.6
Indian Tamil	20.6	1.9	17.6	12.8	28.7	24.5	2.6	20.8	15.2	32.7	15.0	1.6	15.5	8.7	19.9
**Age group (years)**															
18-30	18.1	1.0	16.9	11.7	22.3	21.6	1.7	21.0	14.2	26.5	15.3	1.1	14.4	10.7	19.2
31-40	18.6	0.9	17.1	13.0	22.1	22.8	1.8	20.9	17.0	27.1	17.0	0.9	16.4	12.2	20.0
41-50	18.2	0.7	17.0	13.4	22.0	19.9	1.5	17.4	14.0	25.5	17.4	0.8	16.4	12.9	20.5
51-60	18.8	0.9	16.5	12.0	25.4	23.4	1.6	20.4	15.6	31.3	16.0	1.0	14.4	10.1	19.6
>61	16.6	0.8	15.6	10.5	20.4	19.3	1.4	18.3	14.8	23.7	14.8	1.0	13.0	9.2	18.8
**Educational level**															
No Schooling	15.6	1.2	19.1	10.5	17.0	17.0	2.2	17.2	10.5	22.1	14.6	1.4	15.9	10.1	18.9
Up to 5 years	17.6	0.8	15.4	11.8	20.4	21.9	1.7	18.3	14.1	29.1	15.0	0.8	13.6	10.8	18.8
Up to O/L	17.6	0.6	16.3	12.2	21.0	20.5	1.1	17.4	14.1	26.8	16.2	0.6	15.6	11.4	19.6
Up to A/L	19.9	0.8	18.4	13.9	25.3	22.3	1.3	21.0	15.8	26.9	18.2	1.1	17.7	12.8	22.1
Graduate	17.8	1.6	18.0	10.6	23.0	24.2	2.7	23.3	22.4	27.1	15.3	1.7	13.6	9.8	20.1
**BMI category**															
≤ 18.5 kgm^-2^	16.9	0.9	15.8	11.9	21.2	18.8	1.4	17.2	13.8	23.4	15.4	1.0	14.3	10.6	19.4
>18.5 - ≤ 22.9 kgm^-2^	19.1	0.7	17.1	13.0	22.6	23.0	1.1	20.9	15.6	27.4	15.8	0.8	14.6	11.5	18.8
>23 - ≤ 24.9 kgm^-2^	17.3	1.0	16.2	11.2	22.0	19.1	1.8	16.7	13.2	26.8	16.4	1.1	13.8	10.8	20.8
> 25 - ≤ 27.5 kgm^-2^	18.2	0.7	17.0	13.6	22.4	20.6	1.4	17.2	15.8	26.6	17.4	0.8	16.4	13.1	20.9
≥ 27.5 kgm^-2^	17.2	1.2	15.5	9.3	20.4	23.6	4.4	21.2	11.9	33.2	17.3	0.8	16.4	13.0	20.6

### Sodium

Daily mean sodium intake was 3.26 g and 2.51 g for men and women, respectively (p < 0.05). Dietary sodium intake of Sri Lankan adults according to demographic and BMI categories is shown in Table 7. Mean sodium intake of rural adults was 2.89 g, followed by urban adults (2.73 g). The Estate sector had the lowest intake (2.48 g). Muslims and Sri Lankan Tamils had a higher intake of sodium than Sinhalese and Indian Tamils. With aging, sodium intake declined and the youngest age group recorded the highest intake (3.04 g).

**Table 7:** Sodium intake (mg) of Sri Lankan adults by socio-demographic characteristics

**Characteristics**	**All subjects (n =463)**					**Men (n = 166)**					**Women (n = 297)**				
	**Mean**	**±SE**	**Median**	**Percentiles**		**Mean**	**±SE**	**Median**	**Percentiles**		**Mean**	**±SE**	**Median**	**Percentiles**	
				**25**	**75**				**25**	**75**				**25**	**75**
**Area of residence**															
Urban	2729	102	2509	1835	3411	3100	196	3003	1952	3893	2574	116	2362	1711	3242
Rural	2890	81	2582	2025	3507	3396	155	3190	2448	4211	2605	84	2374	1870	3143
Estate	2477	156	2378	1800	3072	2889	184	2665	2377	3502	1954	200	2036	1359	2350
**Ethnicity**															
Sinhala	2769	61	2523	1934	3391	3155	107	2969	2228	3877	2580	70	2372	1825	3225
Muslim	3012	301	2610	1941	3910	2983	345	3256	2085	3760	3023	407	2469	1612	4023
Sri Lankan Tamil	3306	333	2797	1954	4487	4400	588	4492	2624	5463	2311	176	2189	1803	2859
Indian Tamil	2488	154	2378	1800	3072	2831	184	2598	2144	3467	1997	209	2096	1440	2363
**Age group (years)**															
18-30	3045	145	3071	2186	3519	3436	238	3179	2536	4258	2736	162	2873	1915	3441
31-40	2940	144	2532	2048	3667	3883	311	3856	2379	4669	2584	135	2390	1903	2985
41-50	2778	99	2536	2048	3667	2976	173	2655	2163	3833	2683	120	2480	2023	3245
51-60	2832	162	2448	1817	3441	3188	307	2560	2013	3924	2616	180	2211	1678.	3299
>61	2564	114	2363	1652	3265	3108	187	3106	2290	3822	2201	123	2003	1577	2511
**Educational level**															
No schooling	2290	193	2157	1359	2954	2923	279	2530	2200	3562	1855	206	1740	1203	2302
Up to 5 years	2697	114	2403	1847	3351	2984	169	2772	2198	3813	2521	148	2188	1684	3032
Up to O/L	2825	104	2500	1944	3461	3353	233	3200	2057	3910	2571	99	2371	1903	3031
Up to A/L	2971	113	2715	2122	3437	3300	187	2999	2479	4385	2755	136	2645	1937	3249
Graduate	3046	269	3126	1739	3910	4384	447	4432	3562	5215	2526	241	2663	1506	3408
**BMI category**															
≤ 18.5 kgm^-2^	2580	147	2296	1649	3383	3124	252	2927	2052	4219	2129	129	2054	1541	2793
>18.5 - ≤ 22.9 kgm^2^	3029	114	2665	2069	3622	3464	192	3231	2509	4111	2659	121	2449	1879	3261
>23 - ≤ 24.9 kgm^-2^	2775	150	2486	1914	3241	2896	232	2536	1958	3716	2708	196	2443	1858	3211
> 25 - ≤ 27.5 kgm^-2^	2756	120	2509	1974	3405	3179	119	3213	2278	4126	2596	143	2257	1800	3240
≥ 27.5 kgm^-2^	2615	133	2351	1769	3502	3286	435	3810	2099	4306	2507	135	2255	1660	3287

## Discussion

Although national dietary and nutrition surveys have a number of important functions and can provide much valuable information, Sri Lanka had never conducted a national food consumption survey before, probably due to lack of human and financial resources. This is the first attempt to report energy and macronutrients intakes in a fairly representative sample over the island using updated food composition data. Subject distribution of ethnic groups, area of residence and educational levels closely mirror the national statistics [11].

Differences in calorie consumption were seen according to demographic and BMI categories. Men consume larger portions of foods and are expected to obtain a higher amount of energy than their female counterparts [18]. The intake of energy by Sri Lankan men was found to be higher than that of women by about 350 kcals. Similar differences were reported among Malaysian adults [19] and in Britain the difference was nearly 700 kcal [20]. When compared to people living in urban and rural areas, estate workers are getting the least energy. Lower mean energy intake was reported among Malaysian estate workers [21]. The decline in calorie consumption with age was probably due to reduction in physical activity levels and poor appetite, particularly in older adults. Different energy intakes in ethnic groups may represent their cultural eating habits. For instance, Muslim people tend to have a higher energy intake and eat more fat rich food items compared to Indian Tamils. Up to A/L by education level, energy consumption was gradually increased, this is probably associated with increased purchasing power with higher education status; however, graduate groups may be also aware of health issues associated with excess energy. In developed countries, calorie consumption is inversely associated with education levels [22]. Except for the very obese category, consumption of total energy intake was steadily rising with BMI categories. Under-reporting of food intake by obese subjects is well documented [23].

The total daily intake of protein in Sri Lankan adults is almost half that of the US adults and, among Americans 2/3 of all protein, is derived from animal sources [24]. In contrast, plant sources (rice and pulses) are the main contributors of protein among Sri Lankan adults [10,25]. American men consume over 100 gms of fat daily and for women it is 65 g [26]. Corresponding values for Sri Lankans are 40.5 grams and 31.9 grams. In addition to the amount of fat, the type of fat is crucial for development of diet-related chronic diseases such as cardiovascular disease. Although, sub types of fat are not reported in this analysis, the main lipid source in Sri Lankan diet is coconut milk/oil which is high in saturated fatty acids [27]. Therefore, it is important to conduct further studies to explore the coconut consumption and associated cardiovascular disease risk in this population.

Energy-providing macronutrient proportions could vary in different populations. According to the ranges of population nutrient intake goals recommended by WHO, the percentage of energy from total carbohydrates, fats and proteins should be 55-75%, 15-30% and 10-15%, respectively [28]. British adults consume less than fifty percent of energy (men: 47.7%; women: 48.5%) from carbohydrates, whilst fat intake contributes 35.8% and 34.9% of total energy for men and women respectively. The contribution of protein as an energy source is 16.5% for both sexes [20]. In contrast to western countries, Malaysians get nearly 60% of their energy from carbohydrates, 14% of energy from protein and the rest from fats [19]. In contrast to western countries and some Asian countries, Sri Lankan adults consume proportionally more carbohydrates (>71% of energy) and less fat (<19% of energy) and proteins (<11%). The prevalence of diabetes in Sri Lanka is 11% and one fifth of adults are suffering from diabetes despite low levels of obesity (BMI > 30 = 3.7%). Since the study is cross-sectional in nature, we cannot conclude the association between the relatively larger contribution of energy from carbohydrate and higher prevalence of diabetes/dysglycemia among Sri Lankan adults, in spite of carbohydrates contributing over 70% of energy for both diabetics and non-diabetics. Longitudinal studies assessing the prospective risk of developing diabetes and the proportion of energy derived from macronutrients are needed to fully elucidate an association. A high intake of carbohydrate may lead to hyperinsulinaemia, high serum TAG and low HDL-cholesterol levels and chronic consumption of large carbohydrate meals may cause postprandial hyperglycaemia and hypertriacylglycerolaemia and eventually develop insulin resistance and diabetes [29].

A generous intake of dietary fiber reduces risk of developing many diseases including coronary heart disease, stroke, hypertension, diabetes, obesity, and certain gastrointestinal disorders as well as improving metabolic parameters and immune functions [30]. The definition, method of measuring fiber and recommendations varies in different countries. The backbone of our food composition data is based on USDA. According to US guidelines, the current recommendation is to consume 14 g per every 1000 kcals, therefore using the energy guideline of 2000 kcal/day for women and 2600 kcal/day for men, the recommended daily dietary fiber intake is 28 g/day for adult women and 36 g/day for adult men [31]. Although Sri Lankan adults consume fewer energy compared to US adults, their dietary fiber intake is insufficient according to their calorie intake.

Epidemiological, clinical and animal-experimental evidence showed a direct relationship between dietary electrolyte consumption and blood pressure [32]. Furthermore, clinical trials show that a reduction in salt (NaCl) intake reduces BP levels in normotensive and hypertensive populations and prevents the development of hypertension [32]. Recommended Na intake is maximum of 2.3 g/day [32]. Our findings showed most Sri Lankan adults exceed current recommendations. The high consumption of Sodium may be associated with the epidemic of hypertension (Men: 18.8%; Women: 19.3%) among Sri Lankan adults [33].

This study has several limitations. Sri Lanka has over 20 million inhabitants. Therefore, diet records of a sample of 463 subjects may not represent the eating patterns of the whole population. However, a well-conducted UK NDNS [20] measured the dietary records of 1724 respondents and achieved a lower response rate of 47%. Considering available resources, the high response rate and satisfactory representation of demographic parameters, we believe this is a reasonable sample size. Secondly, 24HDR may not be the best tool to determine habitual diet, because of the non-representative diet and recall bias. However, we selected random 24HDR, which were evenly distributed within weekdays and weekends. Random 24HDR in a large sample has been used in other national surveys in other countries [7]. Thirdly, our findings were limited to energy and selected major macronutrients due to sub quality nutritional information on sub categories of macronutrients and micronutrients of Sri Lankan mixed dishes (Additional file 1). Another limitation is that despite of reports of high alcohol consumption among Sri Lankan men [34], alcohol intake was under-reported in our study (<0.5%). In this survey, low energy reporters (<800 kcal/day) were excluded, therefore exclusion will have biased the data towards higher intakes. Lastly, we did not attempt to correlate energy intake and its adequacy to this population as calorie recommendations may vary with several factors such as gender, age, body weight, body composition and physical activity level.

Acknowledging the limitations of the survey, the present study provides the first national estimates of energy and nutrient intake of the Sri Lanka adult population. It is evident that consumption of high levels of carbohydrate, fat mainly from saturated sources, low protein, low dietary fiber and high levels of sodium may have detrimental effects on health and be related to the current epidemic of NCDs. Unfortunately, current food-based dietary guidelines are based on limited research [25]. Therefore, well-designed and nationally representative studies are needed to explore the association between diet and chronic disease among Sri Lankan adults. Moreover, regular diet and nutrition surveys should be carried out to obtain information on dietary patterns and nutrient intakes and, ideally, periodical monitoring is needed to identify the changing trends in food intake and to assess public responses to dietary recommendations.

## Competing interests

This research received no specific grant from any funding agency in the public, commercial or not-for-profit sectors. The authors declare that they have no financial or non-financial competing interests.

## Authors’ contributions

RJ contributed to the data collection, data analysis and drafted the manuscript. ST analyzed nutrient values. NB, MS, PK and AH were supervisory team members on the project and contributed to study design, interpretation of data and revision of the manuscript. All authors read and approved the final manuscript.

## Supplementary Material

Additional file 1 Selected micronutrient intake among Sri Lankan adults. Click here for file
