# Supplementary material for: Energy and nutrient intakes among Sri Lankan adults
Source: Int Arch Med. 2014 Jul 11;7:34. doi: 10.1186/1755-7682-7-34 (PMC4110527; doi:10.1186/1755-7682-7-34)
Supplement: Additional file 1 — Selected micronutrient intake among Sri Lankan adults. [file 1755-7682-7-34-S1.docx]

| **Micronutrient_unit** | **Total** | | | **Men** | | | **Women** | | |
| --- | --- | --- | --- | --- | --- | --- | --- | --- | --- |
|  | **Mean** | **SD** | **SE** | **Mean** | **SD** | **SE** | **Mean** | **SD** | **SE** |
| Vitamin A_µg | 206.3 | 143.3 | 10.1 | 178.1 | 186.7 | 14.5 | 222.2 | 131.0 | 13.4 |
| Vitamin E_mg | 0.67 | 0.73 | 0.03 | 0.68 | 0.72 | 0.06 | 0.67 | 0.74 | 0.04 |
| Vitamin C_mg | 24.4 | 18.5 | 1.3 | 25.1 | 20.1 | 1.3 | 23.1 | 17.6 | 1.6 |
| Vitamin D_ µg | 4.48 | 10.21 | 0.47 | 4.58 | 10.13 | 0.79 | 4.42 | 10.28 | 0.60 |
| Vitamin B_1__ mg | 1.39 | 0.59 | 0.03 | 1.36 | 0.57 | 0.04 | 1.41 | 0.60 | 0.03 |
| Vitamin B_2__ mg | 1.03 | 0.76 | 0.04 | 1.04 | 0.79 | 0.06 | 1.01 | 0.74 | 0.04 |
| Vitamin B_6__ mg | 1.41 | 3.49 | 0.16 | 1.45 | 2.78 | 0.22 | 1.39 | 3.82 | 0.22 |
| Vitamin B_12__ µg | 1.50 | 2.51 | 0.12 | 1.51 | 2.57 | 0.20 | 1.49 | 2.48 | 0.14 |
| Folic acid_ µg | 29.4 | 20.7 | 0.9 | 30.6 | 20.3 | 1.5 | 28.7 | 20.9 | 1.2 |
| Pottasium_mg | 1444.6 | 552.7 | 25.7 | 1464.6 | 509.3 | 39.5 | 1434.7 | 574.3 | 33.4 |
| Calcium_mg | 420.5 | 234.2 | 10.9 | 430.0 | 223.7 | 17.4 | 403.3 | 239.7 | 13.9 |
| Magnesium_mg | 231.4 | 133.1 | 6.1 | 225.7 | 144.2 | 11.2 | 234.5 | 126.5 | 7.3 |
| Phosphorus_mg | 845.5 | 361.0 | 16.8 | 823.0 | 355.0 | 27.6 | 858.1 | 364.3 | 21.2 |
| Iron_mg | 11.21 | 6.05 | 0.28 | 11.39 | 6.09 | 0.47 | 11.13 | 6.03 | 0.35 |
| Zinc_mg | 9.12 | 6.37 | 0.29 | 9.13 | 6.83 | 0.53 | 9.10 | 6.12 | 0.36 |

**Additional file 1: S**elected micronutrient intake among Sri Lankan adults
